# VHealth-MFusion: a fusion-based multimodal deep learning framework for pneumonia tele-diagnosis in virtual hospital environments

**DOI:** 10.3389/fdgth.2026.1827180

**Published:** 2026-09-02

**Authors:** Sara A. Alsalamah, Albatoul Althenayan, Mariam Almutairi, Shada AlSalamah, Hessah A. Alsalamah, Thamer Nouh, Bassam Mahboub, Laila Salameh, Moteeb Al Moteri, Chang-Tien Lu

**Affiliations:** 1 Department of Computer Science, Virginia Tech, Alexandria, VA, United States; 2 Information Technology Department, College of Computer and Information Sciences, Imam Mohammad Ibn Saud Islamic University, Riyadh, Saudi Arabia; 3 Information Systems Department, College of Computer and Information Science, Imam Mohammad Ibn Saud Islamic University, Riyadh, Saudi Arabia; 4 Information Systems Department, College of Computer and Information Sciences, King Saud University, Riyadh, Saudi Arabia; 5 Computer Engineering Department, College of Engineering, Al Yamamah University, Riyadh, Saudi Arabia; 6 Trauma and Acute Care Surgery Unit, College of Medicine, King Saud University, Riyadh, Saudi Arabia; 7 Department of Pulmonary Medicine, Rashid Hospital, Dubai, United Arab Emirates; 8 Hind Bint Maktoum College of Nursing and Midwifery, Mohammed Bin Rashid University of Medicine and Health Sciences (MBRU), Dubai, United Arab Emirates; 9 Department of Radiology and Medical Imaging, King Khalid University Hospital, King Saud University, Riyadh, Saudi Arabia

**Keywords:** chest X-ray, convolutional neural network, fusion, multimodal learning, pneumonia, tele-diagnosis, virtual hospital

## Abstract

**Introduction:**

Virtual hospital systems support remote triage and diagnosis across distributed healthcare environments by integrating radiographic imaging with structured clinical information for clinical decision making. However, many existing diagnostic frameworks do not fully address real world challenges commonly encountered in scalable virtual healthcare settings. These challenges include severe class imbalance, incomplete clinical attributes, heterogeneous multimodal inputs, and variability in data quality across institutions.

**Methods:**

This study introduces *VHealth-MFusion*, a fusion-based hierarchical multimodal deep learning framework that integrates chest X-ray (CXR) imaging and structured clinical data within a unified convolutional neural network (CNN) and multilayer perceptron (MLP) architecture. Using pneumonia Tele-Diagnosis as a use case, the proposed framework combines radiographic feature extraction through a CNN branch with structured clinical representation learning through an MLP branch, followed by late multimodal fusion for multiclass respiratory disease classification. The framework was evaluated using a hierarchical multimodal dataset containing CXR images across multiple diagnostic categories, including Normal, Bacterial, Adenovirus, Influenza, MERS-CoV, and SARS-CoV-2, together with 632 structured clinical variables comprising demographic information, vital signs, symptoms, and laboratory findings.

**Results:**

Under a controlled dataset-consistent comparison against an established hierarchical multimodal CNN baseline, *VHealth-MFusion* achieved 97.2% classification accuracy, outperforming the baseline accuracy of 95.9%.

**Discussion:**

The findings demonstrate that multimodal integration of radiographic imaging and structured clinical information can improve diagnostic robustness and support more reliable Tele-Diagnosis workflows within virtual hospital environments. Overall, the proposed framework contributes a practically oriented multimodal diagnostic architecture designed to facilitate scalable remote clinical services and informed decision making within digitally enabled care systems where imaging and structured clinical information are jointly available.

## 1 Introduction

The COVID-19 pandemic created an urgent need for accurate, scalable, and remotely deployable diagnostic systems, placing unprecedented pressure on healthcare infrastructures worldwide. Early predictions during the pandemic suggested that virtual healthcare services would play an increasingly important role in post-pandemic healthcare delivery Xtelligent Healthcare Media [1]. Since then, virtual hospital models have gained substantial attention as digitally enabled care environments that support remote diagnosis, monitoring, and clinical decision making across multiple healthcare institutions.

Within such environments, hospitals frequently operate using coordinated hub–spoke structures in which diagnostic expertise is centralized while imaging, testing, and patient monitoring are performed locally. Structured clinical information, including demographic variables, vital signs, laboratory findings, and patient history, is typically collected electronically and integrated into remote care workflows Kumari et al. [2]. This model supports continuity of care beyond physical hospital boundaries while relying heavily on timely, accurate, and interpretable diagnostic support systems.

Artificial intelligence (AI) plays an increasingly important role within these environments by supporting remote triage, early detection, diagnosis, and patient monitoring through integration of heterogeneous clinical information into unified decision-support pipelines. Imaging-based diagnosis represents a particularly attractive component of these workflows because chest X-rays (CXRs) are widely available, cost-effective, and rapidly obtainable across diverse healthcare settings. Previous deep learning studies demonstrated strong diagnostic performance using imaging-only models, while more recent multimodal approaches reported additional improvements by integrating radiographic imaging with structured clinical information.

The integration of imaging and structured clinical variables is especially important in virtual hospital environments because radiographic findings alone may not fully capture physiological severity, symptom progression, or broader patient-specific clinical context associated with respiratory disease diagnosis and remote triage. Consequently, multimodal diagnostic systems capable of jointly modeling radiographic and structured clinical information may better approximate real world clinical decision making processes.

Despite recent advances, many existing multimodal frameworks rely on carefully curated and relatively balanced datasets and do not fully address challenges commonly encountered in real world healthcare environments. These challenges include severe class imbalance, incomplete clinical attributes, heterogeneous multimodal inputs, and variability in data quality across institutions. Such limitations may reduce robustness and hinder practical deployment within scalable virtual and remote healthcare systems.

**Figure 1:**
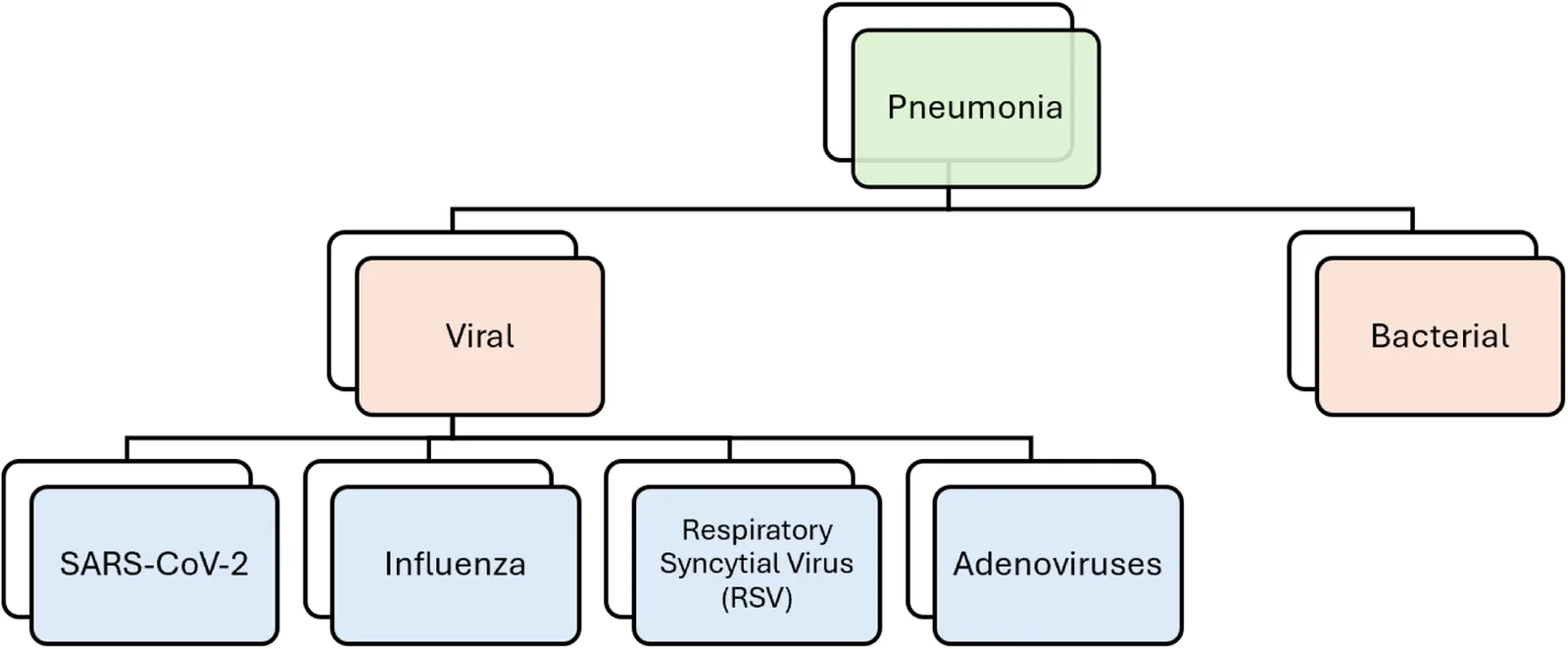
Simplified block diagram of the hierarchical CNN architecture proposed by Althenayan et al. [14], illustrating the layered convolutional structure used for feature extraction and classification. Reproduced from “Proposed hierarchal class structure of pneumonia” by Albatoul S. Althenayan, Shada A. AlSalamah, Sherin Aly, Thamer Nouh, Bassam Mahboub, Laila Salameh, Metab Alkubeyyer and Abdulrahman Mirza, licensed under CC BY 4.0.

To address these challenges, this study introduces *VHealth-MFusion*, a fusion-based hierarchical multimodal deep learning framework that integrates CXR imaging and structured clinical information within a unified architecture for pneumonia diagnosis. Pneumonia, which represents an inflammatory and infectious disease affecting the lungs, is used in this study primarily within the context of COVID-19-related respiratory disease classification. The proposed framework combines a convolutional neural network (CNN) branch for radiographic feature extraction with a multilayer perceptron (MLP) branch for structured clinical data encoding, followed by a joint multimodal fusion layer for classification based on the hierarchical structure of pneumonia (see Figure 1).

This work focuses on developing a robust and practically deployable multimodal diagnostic framework for virtual hospital and remote pneumonia diagnosis settings. The framework is designed to address challenges commonly encountered in real world healthcare environments, including severe class imbalance, incomplete clinical information, heterogeneous multimodal inputs, and constrained deployment conditions.

While the proposed CNN–MLP late-fusion strategy builds upon established multimodal learning principles, the methodological contribution of the framework lies in the integrated imbalance-aware optimization strategy, multimodal preprocessing pipeline, lightweight and computationally efficient architecture, structured handling of sparse clinical variables, and controlled evaluation under highly heterogeneous multimodal healthcare conditions.

Compared with prior hierarchical multimodal approaches, the proposed framework introduces several practical improvements tailored to remote diagnostic workflows, including:

1. Imbalance-aware multimodal optimization combining augmentation, SMOTE, class weighting, dropout regularization, and early stopping under severely skewed disease distributions;
2. Structured preprocessing and feature-reduction strategies for high-dimensional and partially incomplete clinical records;
3. A lightweight multimodal architecture designed to support stable convergence and computational efficiency under constrained healthcare deployment environments; and
4. Controlled dataset-consistent evaluation against an established multimodal baseline under identical experimental conditions.

Collectively, these contributions position the proposed framework as an applied multimodal systems-oriented advancement for virtual hospital diagnostic support rather than as a claim of introducing a fundamentally new deep learning fusion paradigm.

Table 1 summarizes the comparative contribution of the major unimodal and multimodal framework components under the evaluated experimental setting, highlighting the complementary strengths and limitations of each module within the proposed *VHealth-MFusion* architecture.

**Table 1:** Qualitative contribution analysis of unimodal and multimodal framework components under the evaluated experimental setting.

Configuration	Primary strength	Observed limitation
Image-only CNN	Strong radiographic feature extraction from chest X-ray (CXR) images	Sensitive to overlapping radiographic patterns and limited by the absence of contextual clinical information
Clinical-only MLP	Captures demographic, symptom, and laboratory feature relationships	Lacks direct radiographic representation required for robust pneumonia pattern discrimination
CNN–MLP multimodal fusion	Integrates radiographic and structured clinical information for complementary multimodal reasoning	Introduces higher architectural complexity and additional multimodal preprocessing requirements
Imbalance-aware optimization	Enhances minority-class robustness and improves convergence stability under skewed class distributions	Performance for extremely rare classes remains constrained by the limited availability of original samples
Late fusion strategy	Preserves modality-specific feature learning prior to multimodal integration	Does not explicitly model adaptive attention-based weighting between modalities

The remainder of this paper is organized as follows. The Related Work section reviews existing unimodal and multimodal approaches relevant to AI-assisted medical image diagnosis. The Methodology section describes the proposed hierarchical multimodal framework, including the CNN-based imaging branch, the MLP-based structured clinical branch, and the multimodal fusion strategy. The Dataset section presents the dataset composition, preprocessing pipeline, and imbalance-handling procedures. The Results section reports the experimental setup, evaluation metrics, and quantitative findings. The Discussion section provides comparative interpretation of the findings relative to baseline and prior multimodal approaches. Finally, the Conclusion and Future Work section summarizes the main contributions, discusses limitations, and outlines directions for future research.

## 2 Related work

The diagnosis of pneumonia, particularly in the context of COVID-19, has become a major area of research, with numerous studies investigating the use of CXR imaging and deep learning models to improve diagnostic accuracy. Early research primarily focused on image-based approaches that relied exclusively on radiographic information. However, the limitations associated with imaging-only models motivated the development of multimodal learning frameworks that integrate complementary clinical information such as demographic variables, vital signs, symptoms, and laboratory findings to improve diagnostic robustness and clinical contextualization.

This section reviews representative studies spanning image-based diagnostic models, hierarchical and multimodal deep learning approaches, virtual hospital and telemedicine applications, and explainability-oriented clinical AI frameworks. Particular emphasis is placed on the transition from imaging-only analysis toward multimodal diagnostic systems designed to better reflect real world clinical workflows under heterogeneous healthcare conditions.

### 2.1 Image-based COVID-19 diagnosis using CXR

Deep learning-based analysis of CXR images has been widely explored for automated diagnosis of respiratory diseases, including COVID-19. Rajpurkar et al. [3] introduced CheXNet, demonstrating that CNNs could achieve radiologist-level performance in pneumonia detection. Although CheXNet was not designed specifically for COVID-19 diagnosis, it established an important foundation for subsequent CXR-based diagnostic frameworks.

Building upon this direction, Wang and Wong [4] proposed COVID-Net, a CNN architecture specifically designed to capture pneumonia-related radiographic patterns associated with COVID-19 infection. To address limited labeled medical imaging data, several studies subsequently adopted transfer learning strategies using pretrained CNN backbones such as ResNet and EfficientNet. For example, Apostolopoulos and Mpesiana [5] and Hemdan et al. [6] demonstrated that transfer learning approaches can achieve promising diagnostic performance on relatively small CXR datasets. Nevertheless, many imaging-only approaches remain sensitive to dataset bias and often demonstrate limited generalizability across institutions and patient populations.

Widely recognized image-based diagnostic frameworks such as CheXNet Rajpurkar et al. [3] and COVID-Net Wang and Wong [4] established important benchmarks for automated pneumonia and COVID-19 detection using CXR images alone. However, these architectures do not incorporate structured clinical information within a unified multimodal framework. In contrast, the proposed *VHealth-MFusion* framework integrates radiographic imaging with high-dimensional structured clinical variables to support joint multimodal representation learning intended to better reflect real world diagnostic workflows within virtual hospital environments. Consequently, direct numerical comparison with imaging-only frameworks should be interpreted cautiously because of differences in modality scope, dataset composition, and experimental conditions. Nevertheless, these image-based models remain important methodological reference points within the broader landscape of AI-assisted respiratory disease diagnosis.

Recent studies have also demonstrated the effectiveness of EfficientNet-based architectures and transfer learning strategies across multiple medical imaging domains. For example, Billah et al. [7] reported strong performance using EfficientNet-based models for brain tumor MRI classification through efficient feature scaling and transfer learning optimization. Similarly, Das et al. [8] demonstrated that EfficientNet architectures provide strong classification accuracy and computational efficiency for retinal disease classification using fundus imaging. Although these studies focus on different medical imaging modalities, they further reinforce the suitability of EfficientNet-style CNN backbones and transfer learning approaches for extracting clinically meaningful representations from heterogeneous medical imaging data.

### 2.2 Hierarchical and multimodal deep learning approaches

To better approximate real world clinical workflows, hierarchical and multimodal learning approaches have been proposed to integrate radiographic imaging with complementary patient-specific clinical information. Baltrusaitis et al. [9] highlighted that combining multiple modalities can improve robustness and reduce uncertainty when individual data sources are incomplete, noisy, or heterogeneous.

Khan et al. [10] proposed a hierarchical deep learning framework for COVID-19 classification using CXR images. Their architecture decomposed the classification task into multiple stages, improving discrimination between COVID-19, pneumonia, and normal cases. Although the framework achieved approximately 95% classification accuracy, it relied exclusively on imaging data and therefore could not model broader clinical context through structured patient information.

Several studies subsequently explored multimodal deep learning for COVID-19 screening by integrating medical imaging with complementary clinical variables. Chowdhury et al. [11] investigated deep learning approaches for screening viral and COVID-19 pneumonia using CXR images under multiple classification settings. Their reported accuracies generally ranged between 80% and 90% depending on dataset composition and model configuration. Although the study demonstrated the potential of AI-assisted screening, the observed performance variability highlighted ongoing challenges associated with heterogeneous clinical data and multimodal integration.

Similarly, Mei et al. [12] proposed a multimodal AI framework integrating chest CT imaging with non-imaging clinical variables for rapid COVID-19 diagnosis. Their findings demonstrated that multimodal integration can outperform imaging-only models. However, reliance on CT imaging limits direct applicability to CXR-based workflows and large-scale resource-constrained screening environments.

Based on the experimental findings reported by Pereira et al. [13], a hierarchical image-based COVID-19 classification framework achieved an F1-score of approximately 89%. However, the reported performance remained limited by the relatively modest F1-score, restricted dataset size, and reliance on single-modality feature extraction.

To further extend hierarchical multimodal learning, Althenayan et al. [14] combined CXR images with structured clinical information within a hierarchical multimodal CNN framework, achieving approximately 95.9% classification accuracy on a curated multimodal dataset. Although effective, the framework relied on carefully curated subsets and did not fully address severe class imbalance and incomplete clinical variables commonly encountered in real world healthcare environments. In addition, several generative adversarial network (GAN)-based augmentation strategies were explored to generate synthetic minority samples; however, the generated data did not consistently achieve the same level of performance improvement observed with alternative augmentation and imbalance-aware optimization strategies.

### 2.3 Virtual hospitals, telemedicine, and remote diagnostics

The COVID-19 pandemic significantly accelerated adoption of telemedicine and virtual hospital models. Virtual hospitals are generally understood as digitally coordinated care environments that support remote diagnosis, monitoring, and clinical decision making across multiple healthcare institutions. These environments frequently operate using hub-and-spoke structures in which specialized expertise is centralized while imaging, testing, and patient monitoring occur locally through distributed virtual points of care Elrod and Fortenberry [15].

Keesara et al. [16] emphasized the critical role of telemedicine in maintaining continuity of care during the pandemic while reducing exposure risks. Within virtual hospital environments, AI-driven diagnostic systems capable of integrating imaging and electronic health record information are particularly valuable because they better reflect how clinicians combine radiological findings with patient-specific clinical information during remote triage and consultation workflows.

The World Health Organization [17] further highlighted the importance of developing digital healthcare systems that are accurate, interoperable, scalable, and trustworthy to support safe deployment within large-scale remote healthcare environments.

### 2.4 Explainability and safety in clinical AI

Explainability remains a critical requirement for deployment of AI systems within clinical practice. Selvaraju et al. [18] introduced Grad-CAM, a widely adopted visualization technique that highlights image regions contributing to model predictions and supports interpretability of CNN-based diagnostic frameworks.

Similarly, Kelly et al. [19] emphasized that transparency, class-wise performance evaluation, and clear communication of model limitations are essential for achieving meaningful clinical impact and safe deployment of AI-assisted diagnostic systems. These principles are further reinforced by global guidance from the World Health Organization [20], which emphasizes ethical governance, transparency, and responsible deployment of AI technologies within healthcare environments.

Collectively, prior studies demonstrate that hierarchical and multimodal deep learning frameworks show substantial promise for pneumonia diagnosis and AI-assisted respiratory disease screening. Nevertheless, important challenges remain regarding multimodal integration, real world class imbalance, incomplete clinical data, explainability, and deployment scalability within remote and virtual healthcare environments.

Table 2 summarizes representative image-based, hierarchical, multimodal, and explainability-oriented approaches discussed throughout this section, highlighting their input modalities, model architectures, reported performance, and major limitations.

**Table 2:** Comparison of representative related diagnostic approaches.

Study	Modality	Model type	Accuracy	Key limitations
Rajpurkar et al. [3]	CXR Images	CNN (CheXNet)	NA	Not COVID-19 specific
Wang and Wong [4]	CXR Images	CNN (COVID-Net)	∼93%	Image-only; dataset bias
Apostolopoulos and Mpesiana [5]	CXR Images	Transfer learning	∼94%	Small datasets
Hemdan et al. [6]	CXR Images	CNN	∼90%	Limited generalization
Khan et al. [10]	CXR Images	Hierarchical CNN	∼95%	Image-only; no clinical data
Selvaraju et al. [18]	CXR Images	Explainability method (Grad-CAM)	NA	Not a diagnostic model; no multimodal integration
Mei et al. [12]	CT Images & Clinical Data	Multimodal deep learning	∼92%	CT-dependent
Chowdhury et al. [11]	CXR Images & Clinical Data	Multimodal CNN	80%–90%	Basic fusion strategies
Althenayan et al. [14]	CXR Images & Clinical Data	Hierarchical multimodal CNN	95.9%	Curated data; imbalance not fully addressed
**Proposed work**	CXR Images & Clinical Data	CNN & MLP fusion	**97.2%**	Addresses class imbalance, missing data, and multimodal integration challenges

Bold values indicate the results obtained by the proposed VHealth-MFusion framework.

Although direct benchmark comparison across studies remains challenging because of differences in datasets, modalities, and evaluation protocols, inclusion of established image-only frameworks such as CheXNet and COVID-Net provides broader contextual positioning of the proposed multimodal framework within the evolving medical AI literature.

## 3 Methodology

This study builds upon and extends the hierarchical multimodal CNN framework proposed by Althenayan et al. [14], which achieved 95.9% classification accuracy through joint modeling of chest X-ray (CXR) images and structured clinical data. Using the same private multimodal dataset and evaluation protocol, the proposed optimized CNN–MLP fusion framework achieved 97.2% accuracy, representing a 1.3 percentage point improvement over the established multimodal baseline.

Because the underlying multimodal dataset is proprietary and governed by institutional and NDA restrictions, particular emphasis was placed on providing a transparent and reproducible description of the proposed framework. Accordingly, the manuscript explicitly documents the preprocessing pipeline, encoder architectures, multimodal fusion strategy, regularization techniques, optimization settings, training configuration, and evaluation protocol to support methodological reproducibility on alternative multimodal respiratory imaging datasets.

### 3.1 Baseline and comparative framework

The primary baseline for this study is the hierarchical multimodal CNN framework introduced by Althenayan et al. [14]. This work represents the most directly comparable prior approach because it utilizes the same multimodal dataset, including both CXR images and structured clinical variables, within a hierarchical learning framework. To ensure fair and controlled comparison, the present study adopted the same dataset source, preprocessing workflow, class definitions, and train–validation–test partitioning strategy reported in the baseline study. Furthermore, all comparative evaluations were conducted under consistent experimental conditions using aligned preprocessing procedures, dataset partitions, optimization settings, and evaluation metrics whenever applicable.

In addition to the dataset-consistent multimodal baseline comparison, the proposed framework is also contextualized relative to widely recognized image-based architectures such as CheXNet and COVID-Net within the broader medical imaging literature. However, direct numerical comparison across studies should be interpreted cautiously because many multimodal pneumonia diagnosis studies rely on substantially different datasets, imaging modalities, class distributions, preprocessing pipelines, and evaluation protocols. Accordingly, the primary objective of the comparative analysis in this study is to evaluate multimodal integration effectiveness and methodological robustness under controlled dataset-consistent conditions rather than to claim universal superiority over all existing multimodal diagnostic frameworks.

The hierarchical CNN architecture proposed by Khan et al. [10] achieved approximately 90% classification accuracy using CXR images alone. Although this framework provides a validated hierarchical image-processing design, it does not incorporate structured clinical information and therefore does not constitute a multimodal baseline. In the present study, the work of Khan et al. [10] is considered primarily from an architectural perspective as a foundational reference for hierarchical CNN design rather than as a direct performance comparator.

The primary objective of this study is therefore not to introduce a fundamentally new multimodal neural architecture, but rather to investigate how a carefully optimized and practically deployable multimodal framework can improve diagnostic robustness, convergence stability, and classification performance under realistic virtual hospital conditions involving class imbalance, sparse clinical variables, heterogeneous multimodal data, and constrained healthcare deployment environments.

Accordingly, the contribution of the proposed framework should primarily be interpreted from a systems integration, optimization, and healthcare applicability perspective rather than solely from the introduction of a completely novel deep learning fusion mechanism. Specifically, the proposed framework improves multimodal fusion effectiveness and robustness through an optimized CNN–MLP architecture combined with imbalance-aware training strategies, thereby enhancing integration between heterogeneous modalities while maintaining architectural consistency with prior hierarchical multimodal CNN approaches.

Overall, the present study provides a direct dataset-consistent methodological improvement over the established multimodal baseline of Althenayan et al. [14], while appropriately positioning earlier image-only work by Khan et al. [10] as foundational but non-comparative.

### 3.2 Proposed fusion-based hierarchical multimodal framework

To address the limitations associated with isolated unimodal learning, a fusion-based hierarchical multimodal learning framework was developed to jointly model radiographic imaging and structured clinical information for respiratory disease diagnosis.

To mitigate the effect of severe class imbalance, the training pipeline incorporated SMOTE-based oversampling for minority classes together with image augmentation strategies including horizontal flipping, small rotations, and contrast adjustment. In addition, class-weighted optimization was applied during training to promote balanced loss contribution across disease categories.

**Figure 2:**
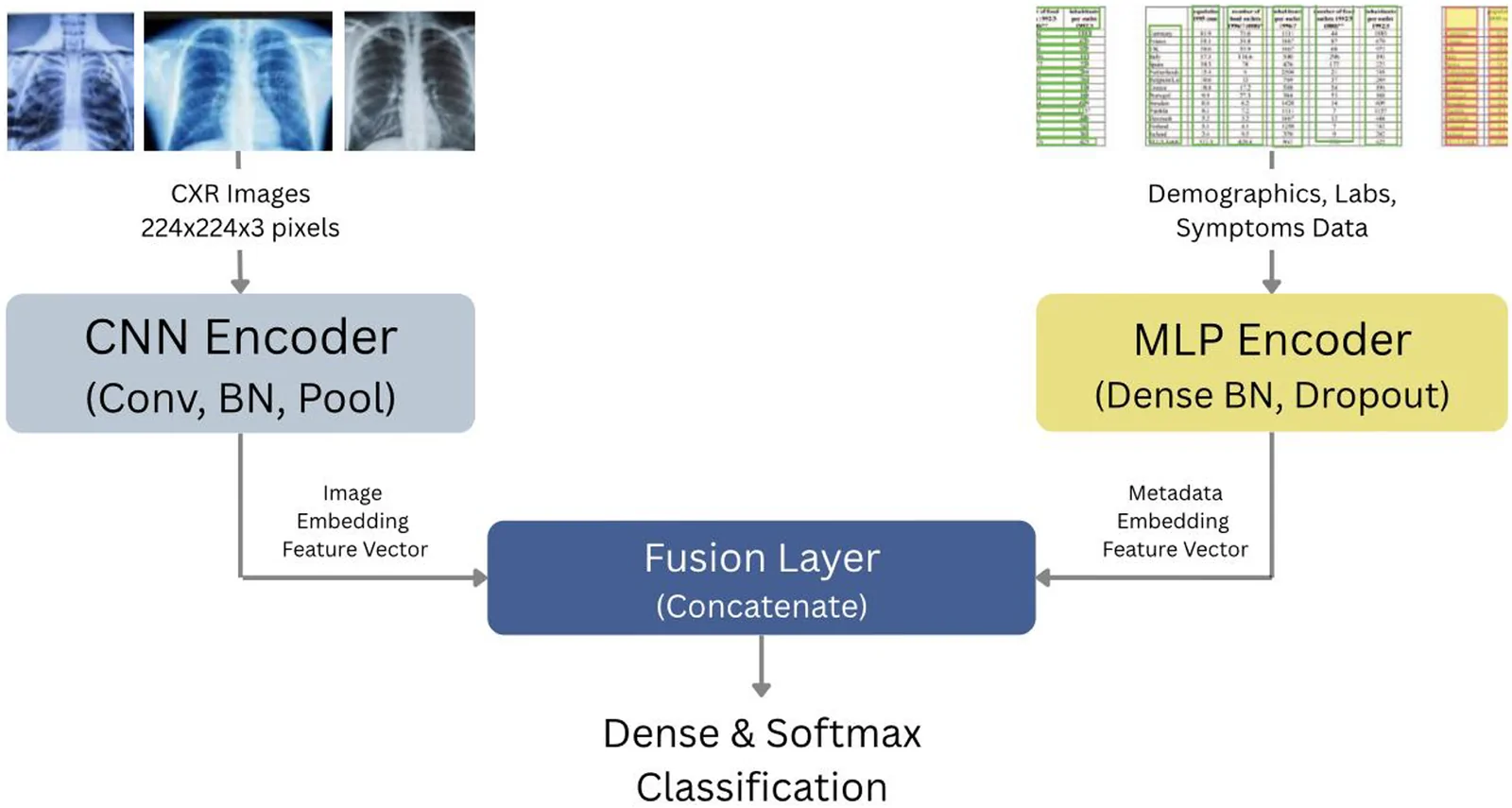
Proposed fusion-based hierarchical multimodal framework for pneumonia diagnosis, integrating radiographic imaging and structured clinical information through a unified multimodal fusion and classification pipeline.

Figure 2 illustrates the overall architecture of the proposed multimodal framework.

#### 3.2.1 CNN branch (image encoder)

The CNN branch processes preprocessed CXR images resized to 224×224 pixels and normalized to the [0,1] range. A lightweight hierarchical CNN architecture was adopted to extract radiographic representations relevant to pneumonia diagnosis while maintaining computational efficiency and stable multimodal optimization.

The CNN encoder consists of three sequential convolutional blocks. Each block includes convolutional operations followed by batch normalization, Rectified Linear Unit (ReLU) activation, and max-pooling layers to progressively learn hierarchical spatial representations while reducing feature dimensionality. These convolutional operations capture both low-level and high-level radiographic characteristics, including pulmonary opacities, infiltrates, texture abnormalities, and lung pattern variations associated with respiratory infection.

The convolutional operation is mathematically represented as shown in Equation 1:

$$F_{l}=\sigma (W_{l}*X_{l}+b_{l})$$ (1)

where $X_{l}$ denotes the input feature map at layer l, $W_{l}$ represents the convolution kernel weights, $b_{l}$ is the bias term, ∗ denotes convolution, and σ(⋅) represents the nonlinear activation function.

Following the final convolutional block, Global Average Pooling (GAP) is applied to transform the spatial feature maps into a compact latent radiographic embedding, as shown in Equation 2:

$$z_{\mathrm{img}}=\mathrm{GAP}(F_{L})$$ (2)

where $F_{L}$ denotes the final convolutional feature representation and $z_{\mathrm{img}}$ represents the extracted radiographic embedding used for multimodal fusion.

Dropout regularization is subsequently applied to reduce overfitting and improve generalization stability prior to multimodal integration.

A lightweight custom CNN architecture was selected to balance representational capacity, computational efficiency, and training stability within the current multimodal experimental setting. Because the framework jointly processes imaging and high-dimensional structured clinical variables, employing a lightweight image encoder reduced computational overhead and facilitated stable multimodal optimization under limited dataset conditions. In addition, lighter architectures may reduce overfitting risk relative to substantially deeper pretrained networks when training data are relatively constrained and imbalanced.

The CNN encoder was trained directly on the multimodal pneumonia dataset rather than initialized using transfer learning from a large external pretrained pneumonia-specific model. The primary objective of this study was to evaluate the effectiveness of the proposed multimodal fusion framework itself under controlled dataset-consistent conditions. Nevertheless, pretrained architectures such as ResNet and EfficientNet remain promising directions for future investigation and may further improve radiographic feature generalization across larger multicenter datasets.

#### 3.2.2 MLP branch (tabular encoder)

The MLP branch processes structured clinical variables including demographic information, symptoms, physiological observations, laboratory measurements, and outcome-related attributes. Following preprocessing, feature reduction, normalization, and missing-value imputation, the structured clinical input vector is passed through a three-layer multilayer perceptron architecture composed of fully connected dense layers.

Each dense layer performs nonlinear transformation of the structured clinical feature space according to Equation 3:

$$h_{l}=\phi (W_{l}h_{l-1}+b_{l})$$ (3)

where $h_{l-1}$ denotes the input representation from the previous layer, $W_{l}$ and $b_{l}$ represent trainable weights and bias parameters, and ϕ(⋅) denotes the nonlinear activation function.

Batch normalization and dropout regularization are applied after each dense layer to improve convergence stability and reduce overfitting within the high-dimensional clinical feature space. The MLP progressively transforms structured clinical variables into a compact latent clinical representation, as described in Equation 4:

$$z_{\mathrm{clinical}}=\mathrm{MLP}(x_{\mathrm{clinical}})$$ (4)

where $x_{\mathrm{clinical}}$ represents the preprocessed structured clinical feature vector and $z_{\mathrm{clinical}}$ denotes the latent clinical embedding used for multimodal fusion.

Importantly, the multilayer dense architecture enables the framework to learn nonlinear feature interactions among symptoms, laboratory findings, demographic characteristics, and physiological indicators. Rather than treating each clinical variable independently, the network captures multivariate patterns in which combinations of clinical attributes contribute jointly to classification decisions. These learned clinical feature representations are subsequently fused with radiographic feature embeddings, enabling the model to leverage complementary information from both modalities during the final classification process.

The three-layer MLP architecture was selected as a balance between representational capacity, training stability, computational efficiency, and dataset scale. Preliminary exploration of deeper architectures substantially increased model complexity and training instability without producing consistent improvements in validation performance. Given the relatively limited dataset size and the presence of imbalance and sparsity within the structured clinical variables, deeper architectures also demonstrated greater susceptibility to overfitting. In contrast, the adopted three-layer design demonstrated stable convergence behavior and effective integration with the CNN branch while maintaining strong multimodal classification performance.

To further reduce redundancy within the high-dimensional clinical feature space, preprocessing incorporated feature-reduction strategies targeting highly correlated and low-information variables. This reduced unnecessary feature overlap while preserving clinically relevant information for multimodal fusion.

#### 3.2.3 Fusion and classification layer

The multimodal fusion stage integrates the latent radiographic embedding generated by the CNN branch with the latent structured clinical embedding generated by the MLP branch. A late-fusion strategy was adopted because imaging and structured clinical variables represent heterogeneous modalities with distinct statistical and representational characteristics.

The latent feature representations are concatenated into a unified multimodal feature vector, as shown in Equation 5:

$$z_{\mathrm{fusion}}=[z_{\mathrm{img}};z_{\mathrm{clinical}}]$$ (5)

where [⋅;⋅] denotes feature concatenation.

The fused multimodal representation is subsequently passed through fully connected dense layers with nonlinear activations and dropout regularization prior to final classification, as defined in Equation 6:

$$y=\mathrm{Softmax}(W_{\;f}z_{\mathrm{fusion}}+b_{\;f})$$ (6)

where $W_{\;f}$ and $b_{\;f}$ denote trainable classification parameters and y represents the predicted probability distribution across respiratory disease categories.

This late-fusion strategy enables the CNN branch to independently learn radiographic representations while allowing the MLP branch to independently model structured clinical relationships prior to multimodal integration. The resulting fused latent representation supports joint multimodal reasoning across imaging and patient-specific clinical information within virtual hospital diagnostic workflows.

The current framework does not explicitly implement adaptive attention or dynamic weighting mechanisms that assign variable importance to specific clinical variables based on radiographic findings. Instead, multimodal interactions are learned implicitly within the fused latent representation space generated during multimodal training. Future work will investigate attention-based multimodal fusion and explainability-oriented techniques such as SHAP and Grad-CAM to better quantify the contribution of specific radiographic patterns and structured clinical variables during diagnostic prediction.

#### 3.2.4 Integrated multimodal architecture

**Figure 3:**
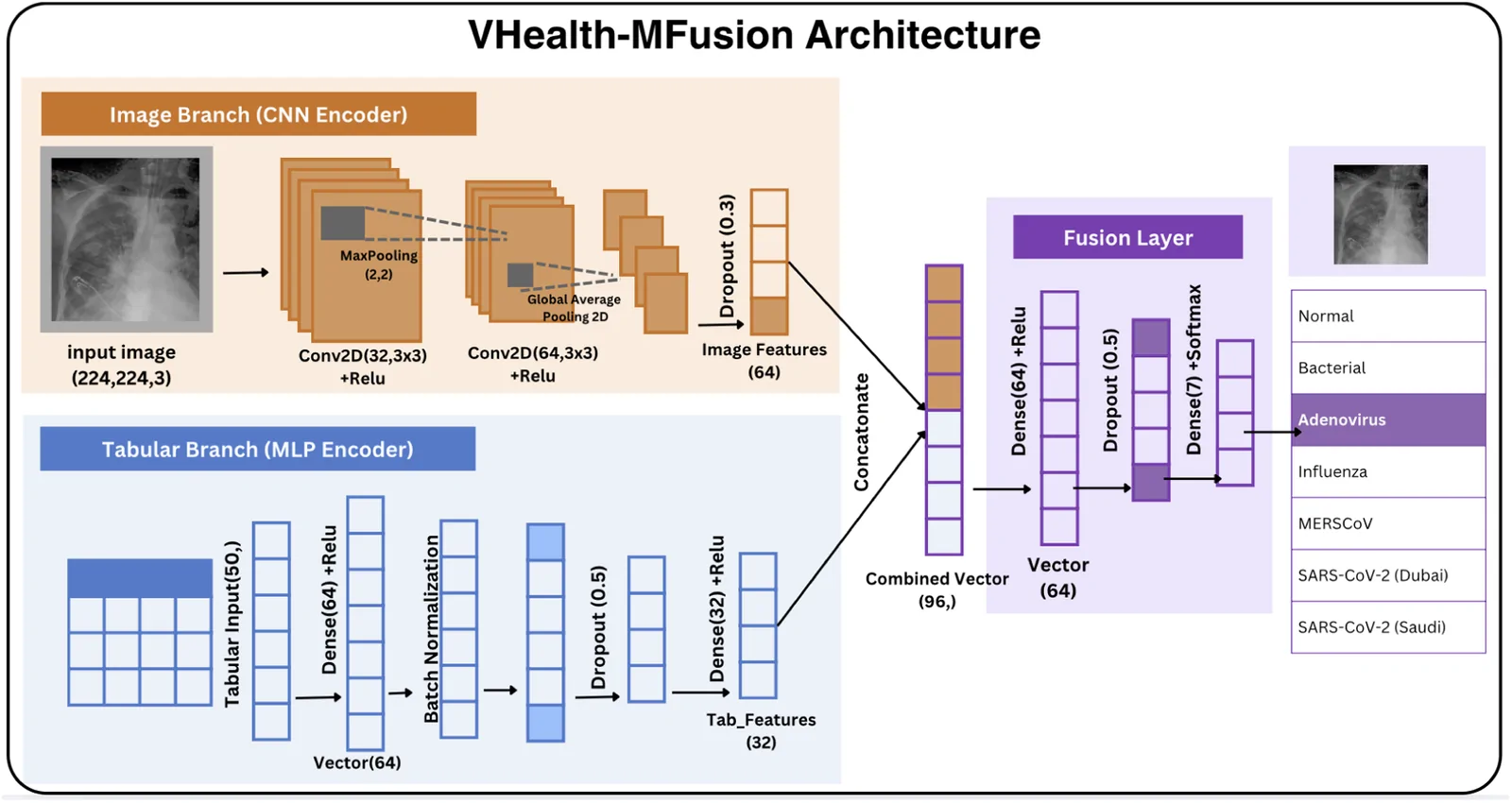
Architectural illustration of the proposed multimodal framework, showing the CNN-based image branch, the MLP-based tabular branch, and their integration through a feature fusion layer for final classification.

Figure 3 illustrates the integrated multimodal architecture, consisting of a CNN-based image encoder for radiographic feature extraction, an MLP-based tabular encoder for structured clinical representation learning, and a late-fusion layer for joint multimodal classification.

### 3.3 Training strategy

A training strategy explicitly designed to improve generalization and reduce overfitting within the high-dimensional multimodal feature space was adopted. This strategy incorporated dropout regularization, early stopping, class-weighted learning, SMOTE-based minority oversampling, and image augmentation techniques including horizontal flipping, small rotations, and contrast adjustment.

Dropout regularization with a rate of 0.5 was applied within both the CNN and MLP branches to randomly deactivate subsets of neurons during training, thereby reducing feature co-adaptation and discouraging memorization of patient-specific noise or spurious correlations. Early stopping was additionally employed to monitor validation performance and terminate training once validation loss no longer improved.

Missing structured clinical values were handled using statistical imputation strategies prior to multimodal training, including median imputation for continuous variables and mode-based imputation for categorical variables.

Although SMOTE was applied to mitigate class imbalance for minority disease categories, multiple complementary regularization strategies were incorporated to reduce the risk of overfitting to synthetic minority samples. Specifically, dropout regularization, augmentation, early stopping, and class-weighted optimization were jointly applied during training to improve generalization stability.

Furthermore, the training and validation accuracy/loss curves demonstrated stable convergence behavior without substantial divergence between training and validation performance, suggesting that the framework generalized beyond synthetic oversampled instances rather than merely memorizing minority-class samples. In addition, minority-class performance remained relatively balanced across precision, recall, and F1-score metrics instead of exhibiting artificially inflated training performance alone. Collectively, these observations support the stability and robustness of the proposed multimodal learning strategy under highly imbalanced dataset conditions.

### 3.4 Training configuration and hyperparameters

Table 3 summarizes the key hyperparameters and training configuration settings used for the proposed CNN–MLP multimodal framework.

**Table 3:** Training hyperparameters and configuration settings for the proposed CNN–MLP multimodal framework.

Hyperparameter	Value
Optimizer	Adam
Learning rate	$1\times 10^{-4}$
Loss function	Categorical cross-entropy
Batch size	32
Epochs	50 (early stopping, patience=5)
Class weighting	Yes
Evaluation metrics	Accuracy, Precision, Recall

To improve experimental transparency and comparative fairness, all evaluated models within the controlled comparative framework were trained and assessed under aligned preprocessing procedures, equivalent dataset partitions, and standardized evaluation metrics whenever supported by the corresponding architecture. All preprocessing, training, and evaluation stages were additionally implemented using modular pipelines with consistent parameter settings across experiments.

The framework was trained using the Adam optimizer with categorical cross-entropy loss together with class-weighted learning, dropout regularization, early stopping, and augmentation-aware preprocessing under identical evaluation conditions across comparative experiments.

### 3.5 Improvements over prior work

Compared with Althenayan et al. [14], the proposed methodology introduces several important improvements:

1. **Optimized Multimodal Fusion:** The proposed framework improves integration between radiographic imaging and structured clinical variables through an optimized CNN–MLP multimodal fusion strategy designed for heterogeneous healthcare data.
2. **Imbalance-Aware Training:** Class-weighted optimization, SMOTE-based oversampling, augmentation, and regularization strategies were incorporated to improve robustness under severe class imbalance conditions.
3. **Modular and Reproducible Architecture:** The proposed framework maintains a modular multimodal architecture that supports reproducibility while achieving improved diagnostic performance relative to the baseline framework.
4. **Improved Multimodal Stability:** The combined use of multimodal fusion, regularization, feature reduction, and controlled optimization contributed to more stable convergence and improved generalization under heterogeneous clinical conditions.

By combining CNN-based radiographic analysis with MLP-based structured clinical representation learning, the proposed multimodal framework improves diagnostic robustness and multimodal integration effectiveness relative to the hierarchical multimodal CNN baseline. These improvements support more reliable multimodal learning under imbalanced healthcare conditions while maintaining practical applicability for remote and virtual hospital diagnostic workflows.

## 4 Dataset

The dataset used in this study integrates radiographic imaging and structured clinical information to support multimodal learning for pneumonia diagnosis. The imaging component consists of CXR scans categorized into multiple respiratory disease classes, while the structured clinical component contains demographic variables, symptoms, physiological observations, laboratory findings, and outcome-related information associated with the corresponding patient records. This is the same curated multimodal dataset employed by Althenayan et al. [14], enabling controlled dataset-consistent comparison between the baseline and proposed multimodal frameworks.

The diagnostic labels and disease categories were inherited from the original curated multimodal dataset introduced by Althenayan et al. [14]. Because the dataset was obtained under institutional agreements and NDA restrictions, detailed annotation workflow information, including the number of contributing clinical experts and institution-specific labeling procedures, cannot be publicly disclosed within the current manuscript. Nevertheless, the dataset was originally curated for clinical diagnostic research purposes and was used consistently across both the baseline and proposed multimodal evaluation frameworks.

Although the dataset includes heterogeneous respiratory disease categories and multimodal clinical information collected from multiple healthcare sources within the original dataset composition, the current study evaluates the proposed framework under controlled experimental conditions using a single curated multimodal dataset. Therefore, the reported findings should primarily be interpreted as controlled framework-level validation rather than definitive evidence of universal clinical generalizability across independent healthcare institutions or geographically distinct populations.

### 4.1 Image data

**Figure 4:**
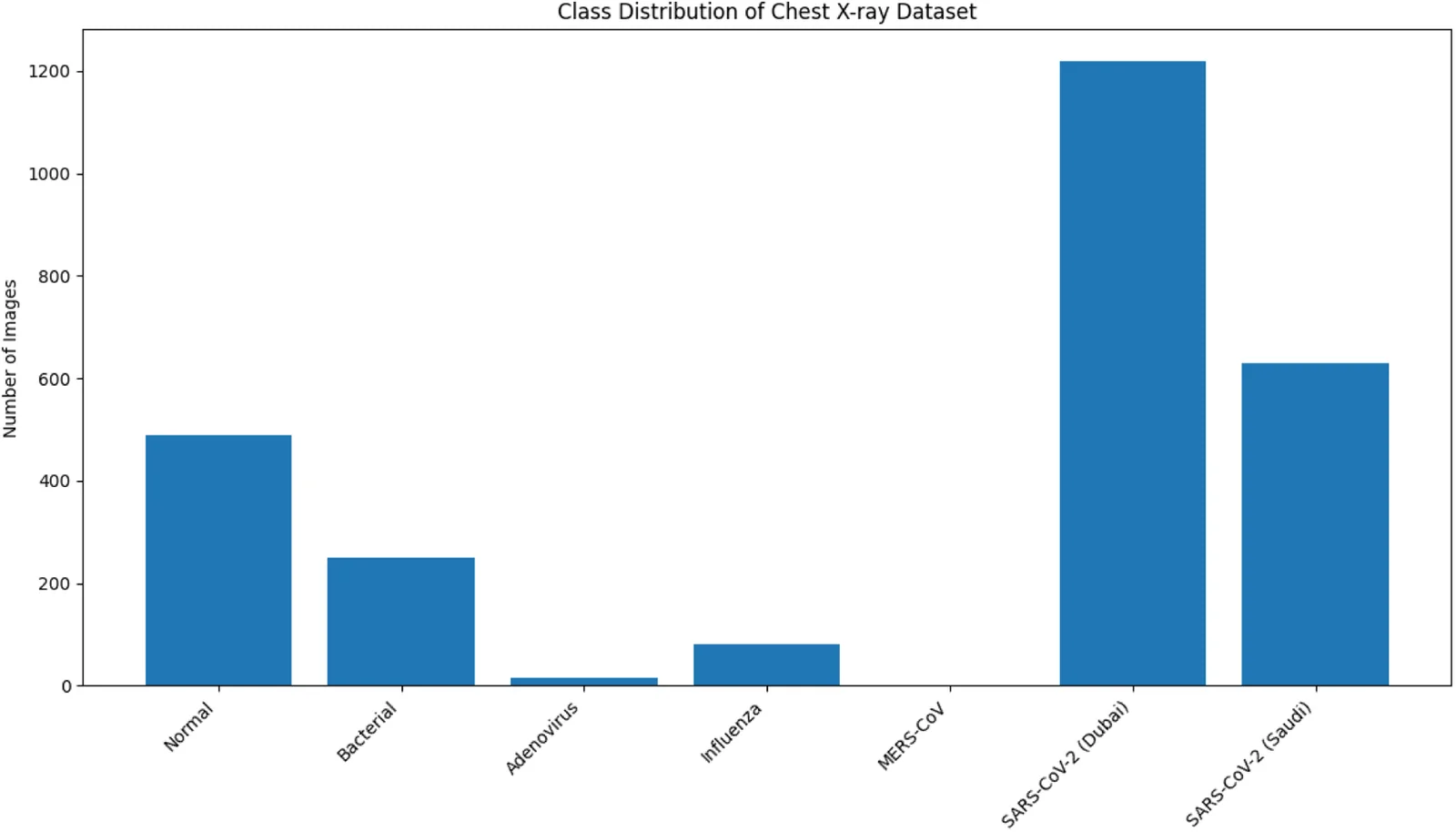
Distribution of CXR images across diagnostic categories. The dataset is highly imbalanced, with SARS-CoV-2 Dubai and Saudi subsets dominating the distribution, while rare classes such as MERS-CoV and Adenovirus remain underrepresented.

The imaging component consists of CXR scans categorized into multiple diagnostic classes, including Normal, Bacterial, Adenovirus, Influenza, MERS-CoV, and SARS-CoV-2 (Dubai and Saudi subsets). As shown in Table 4 and Figure 4, the dataset is highly imbalanced, with the SARS-CoV-2 Dubai and Saudi categories dominating the dataset distribution, while rare conditions such as MERS-CoV and Adenovirus contain only a limited number of samples.

**Table 4:** Distribution of CXR images across diagnostic categories within the dataset.

Class	Number of images
Normal	490
Bacterial	249
Adenovirus	16
Influenza	81
MERS-CoV	1
SARS-CoV-2 (Dubai)	1,219
SARS-CoV-2 (Saudi)	630

In particular, the MERS-CoV category contains only a single original image and is therefore retained primarily as an exploratory proof-of-concept category within the hierarchical disease taxonomy rather than as a statistically reliable standalone diagnostic class. Consequently, the present study does not claim clinically generalizable or statistically robust performance for MERS-CoV classification. The inclusion of this category is intended to preserve the clinical heterogeneity and disease taxonomy of the original multimodal dataset used for comparative evaluation.

### 4.2 Example CXR images

**Figure 5:**
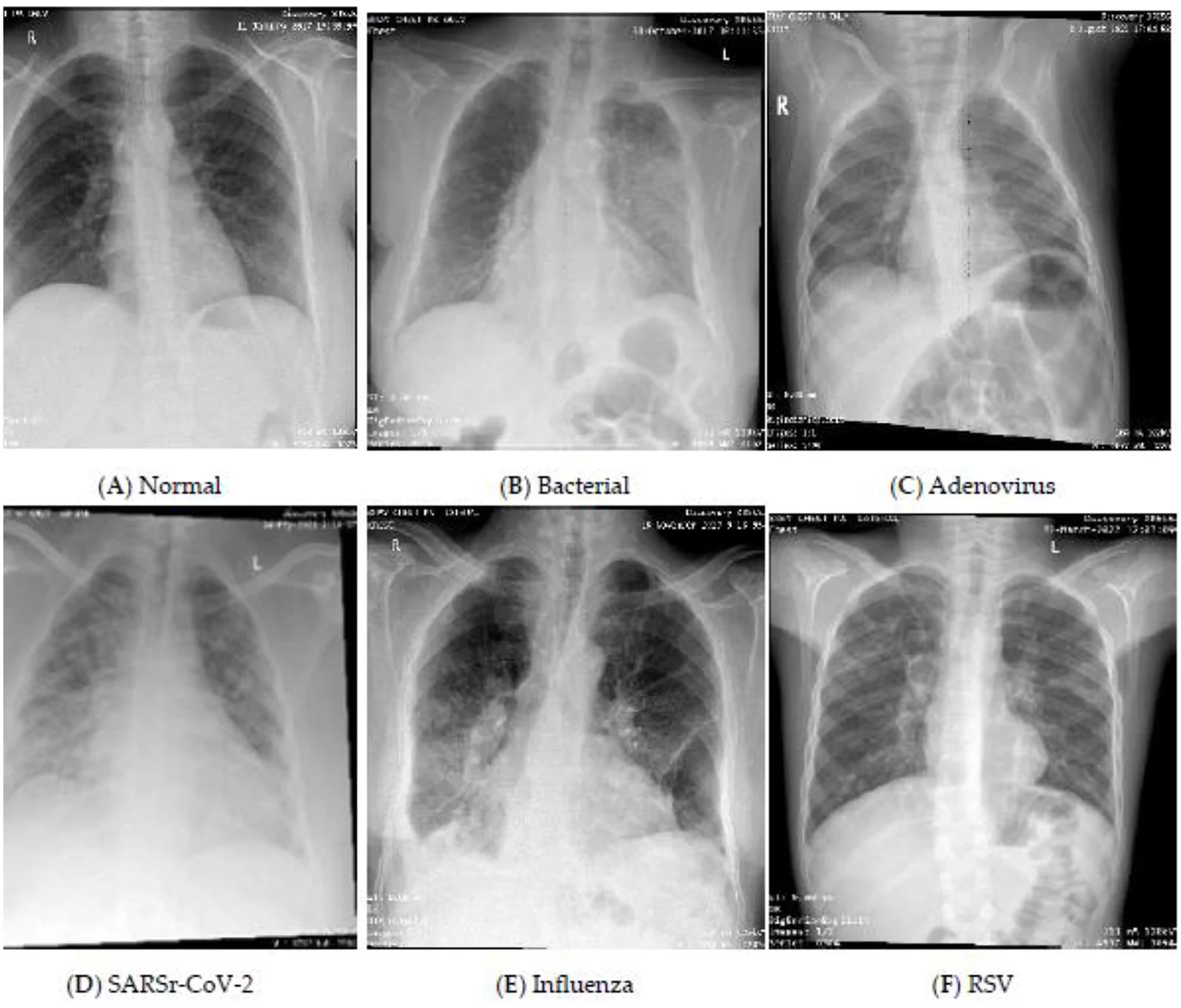
Representative chest X-ray images of different respiratory conditions: **(A)** Normal, **(B)** Bacterial pneumonia, **(C)** Adenovirus, **(D)** SARS-CoV-2, **(E)** Influenza, and **(F)** Respiratory Syncytial Virus (RSV). The images illustrate characteristic radiographic variations observed across the different diagnostic categories [14]. Reproduced from “CXR samples of the dataset” by Albatoul S. Althenayan, Shada A. AlSalamah, Sherin Aly, Thamer Nouh, Bassam Mahboub, Laila Salameh, Metab Alkubeyyer and Abdulrahman Mirza, licensed under CC BY.

Representative CXR samples are shown in Figure 5 to illustrate the visual variability across diagnostic categories and the inherent challenges associated with distinguishing overlapping respiratory disease patterns.

### 4.3 Structured clinical data

In addition to radiographic imaging, the dataset includes structured clinical information containing 632 variables associated with the corresponding patient records. These variables include demographic information, clinical symptoms, physiological observations, laboratory measurements, and outcome-related attributes. The structured clinical data provide complementary contextual information that supports radiographic interpretation by capturing patient-specific physiological and clinical conditions that may not be visually apparent from CXR images alone.

Examples of clinically relevant variables include age, sex, fever, oxygen saturation, respiratory symptoms, inflammatory laboratory markers, and hospitalization-related indicators. Within real world virtual hospital workflows, clinicians typically interpret radiographic findings together with patient history, laboratory results, and physiological observations during pneumonia diagnosis and triage. Accordingly, the proposed multimodal framework integrates both imaging and structured clinical information to better approximate practical remote diagnostic decision making processes.

The structured clinical dataset additionally includes demographic variables such as age, sex, and geographic region as part of the multimodal feature space to support clinically contextualized prediction. However, because the dataset is governed by institutional privacy restrictions and NDA limitations, detailed patient-level demographic distributions cannot be publicly disclosed. Nevertheless, demographic variables were retained during training and evaluation to ensure that the framework learned from heterogeneous patient characteristics rather than relying exclusively on imaging features alone.

### 4.4 Train, validation, and test split

Stratified sampling was applied to divide the dataset into training, validation, and testing subsets while preserving class proportions as much as possible. However, due to the extreme imbalance of certain minority categories, particularly MERS-CoV, some classes could not be represented across all dataset partitions because only a single original sample was available.

In the current partitioning scheme, the MERS-CoV sample appears exclusively within the training set and is absent from both the validation and test subsets. Consequently, MERS-CoV-specific validation or test metrics cannot be reported and should not be interpreted as statistically evaluable under the present experimental setting. The dataset split statistics are summarized in Table 5.

**Table 5:** Train, validation, and test partitioning across diagnostic categories.

Class	Train	Validation	Test
Normal	343	74	73
Bacterial	174	38	37
Adenovirus	11	2	3
Influenza	57	12	12
SARS-CoV-2 (Dubai)	853	183	183
SARS-CoV-2 (Saudi)	441	95	94

### 4.5 Dataset challenges

The dataset presents several challenges common to real world multimodal healthcare data. The most significant challenge is the extreme class imbalance, where some diagnostic categories contain only a limited number of samples. In addition, the structured clinical branch contains high-dimensional data with sparse and partially missing values. These challenges motivated the design of the preprocessing pipeline and informed the architecture and regularization strategies adopted within the proposed multimodal framework.

### 4.6 Preprocessing

Preprocessing was performed separately for the imaging and structured clinical branches to ensure both modalities were standardized and appropriately aligned prior to multimodal fusion.

#### 4.6.1 Image preprocessing

**Figure 6:**
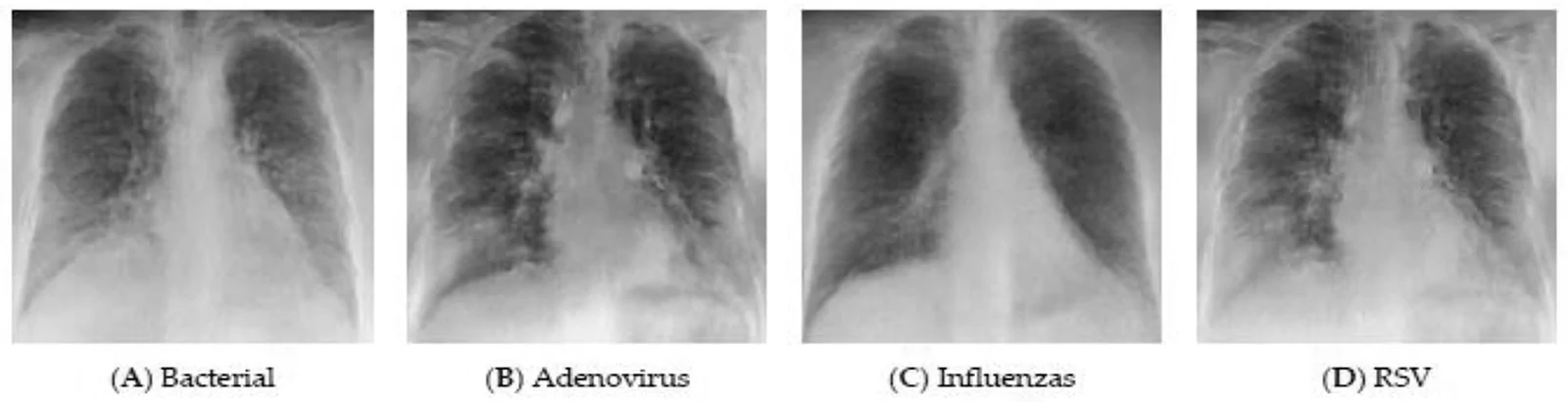
Representative chest X-ray examples highlighting radiographic manifestations of respiratory infections: **(A)** Bacterial pneumonia, **(B)** Adenovirus, **(C)** Influenza, and **(D)** Respiratory Syncytial Virus (RSV). The images demonstrate differences in lung opacity patterns associated with each condition [14]. Reproduced from “Samples of synthetic CXR images” by Albatoul S. Althenayan, Shada A. AlSalamah, Sherin Aly, Thamer Nouh, Bassam Mahboub, Laila Salameh, Metab Alkubeyyer and Abdulrahman Mirza, licensed under CC BY.

All CXR images were resized to 224×224 pixels and normalized to the [0,1] range to ensure consistency across the imaging branch. To reduce overfitting and partially mitigate class imbalance, several augmentation strategies were applied, including random horizontal flipping, small rotations of up to $\pm 10^{\circ }$, and contrast adjustments. These augmentations preserve medically relevant radiographic structures while increasing training diversity. Representative examples of the preprocessed CXR images are shown in Figure 6.

#### 4.6.2 Tabular data preprocessing

The structured clinical branch included demographic, clinical, and laboratory variables containing sparse, incomplete, and partially missing information. Accordingly, a preprocessing pipeline was applied prior to multimodal training.

Continuous numerical variables were imputed using median-value replacement to reduce sensitivity to outliers and maintain robustness under heterogeneous clinical distributions. Categorical variables were completed using mode-based imputation, where missing entries were replaced using the most frequently occurring category within the corresponding feature. Following imputation, continuous variables were standardized and categorical variables were numerically encoded to ensure compatibility with the MLP branch.

To improve computational efficiency and reduce redundancy within the high-dimensional feature space, highly sparse, low-variance, and strongly correlated variables were identified and removed during preprocessing. This strategy helped reduce noise, stabilize optimization, and mitigate excessive feature overlap while preserving clinically relevant information for multimodal fusion. All preprocessing and imputation procedures were applied consistently across the dataset prior to model training and evaluation.

## 5 Results

The proposed hierarchical multimodal fusion framework demonstrated strong diagnostic performance and improved multimodal learning stability under highly heterogeneous and imbalanced healthcare conditions. The framework was evaluated using the same multimodal dataset, class definitions, preprocessing workflow, and train–validation–test split configuration used by Althenayan et al. [14] to ensure fair and controlled comparative evaluation.

The baseline hierarchical multimodal CNN reported by Althenayan et al. [14] achieved a classification accuracy of 95.9% on the same dataset. In comparison, the proposed CNN–MLP multimodal fusion framework achieved a validation accuracy of 97.2%. Importantly, the observed improvement was not limited to overall accuracy alone, but was also reflected across complementary evaluation metrics including precision, recall, sensitivity, F1-score, confusion matrix behavior, and validation convergence stability.

To reduce the likelihood that the observed performance improvement resulted primarily from favorable dataset partitioning, all comparative experiments were conducted under identical experimental conditions using the same dataset source, preprocessing procedures, class definitions, evaluation metrics, and train–validation–test partitions. The proposed framework consistently demonstrated improved multimodal performance trends across multiple complementary evaluation measures rather than isolated gains in a single metric.

Although formal repeated cross-validation, confidence interval estimation, and statistical hypothesis testing were not performed because of the computational complexity and dataset constraints associated with multimodal deep learning training, the model demonstrated relatively stable validation behavior throughout optimization. Furthermore, the observed improvements remained consistent under the controlled experimental setting used throughout the study, supporting the reliability of the reported multimodal performance trends.

### 5.1 Training and validation performance

**Figure 7:**
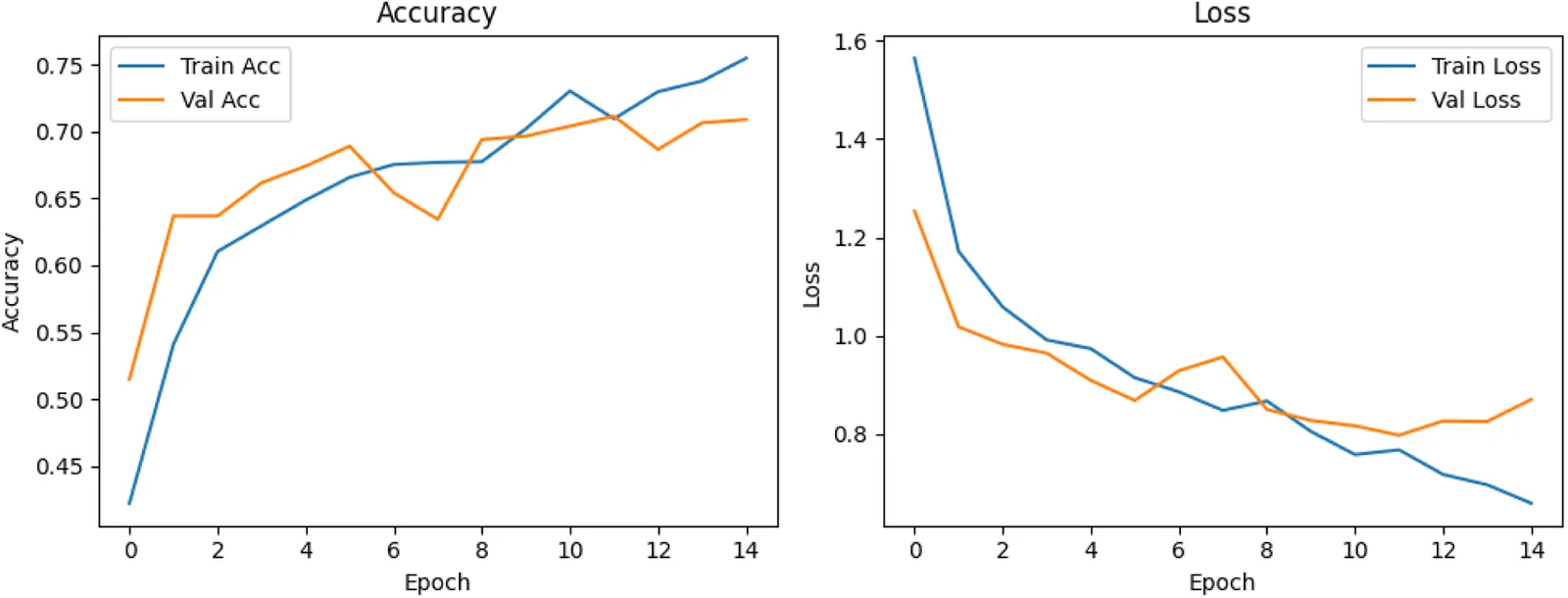
Training and validation accuracy and loss curves over successive training epochs.

The training curves demonstrated stable optimization behavior throughout the training process, with training accuracy remaining closely aligned with validation accuracy across epochs. Early stopping and dropout regularization contributed to limiting overfitting within the high-dimensional multimodal feature space, while both training and validation loss decreased consistently during optimization. Figure 7 illustrates the training and validation accuracy and loss curves.

The relatively close alignment between training and validation accuracy suggests that the framework maintained stable generalization behavior during learning. In addition, validation loss did not exhibit substantial late-stage divergence or instability, further supporting the effectiveness of the adopted regularization strategies in stabilizing multimodal learning.

### 5.2 Confusion matrix analysis

**Figure 8:**
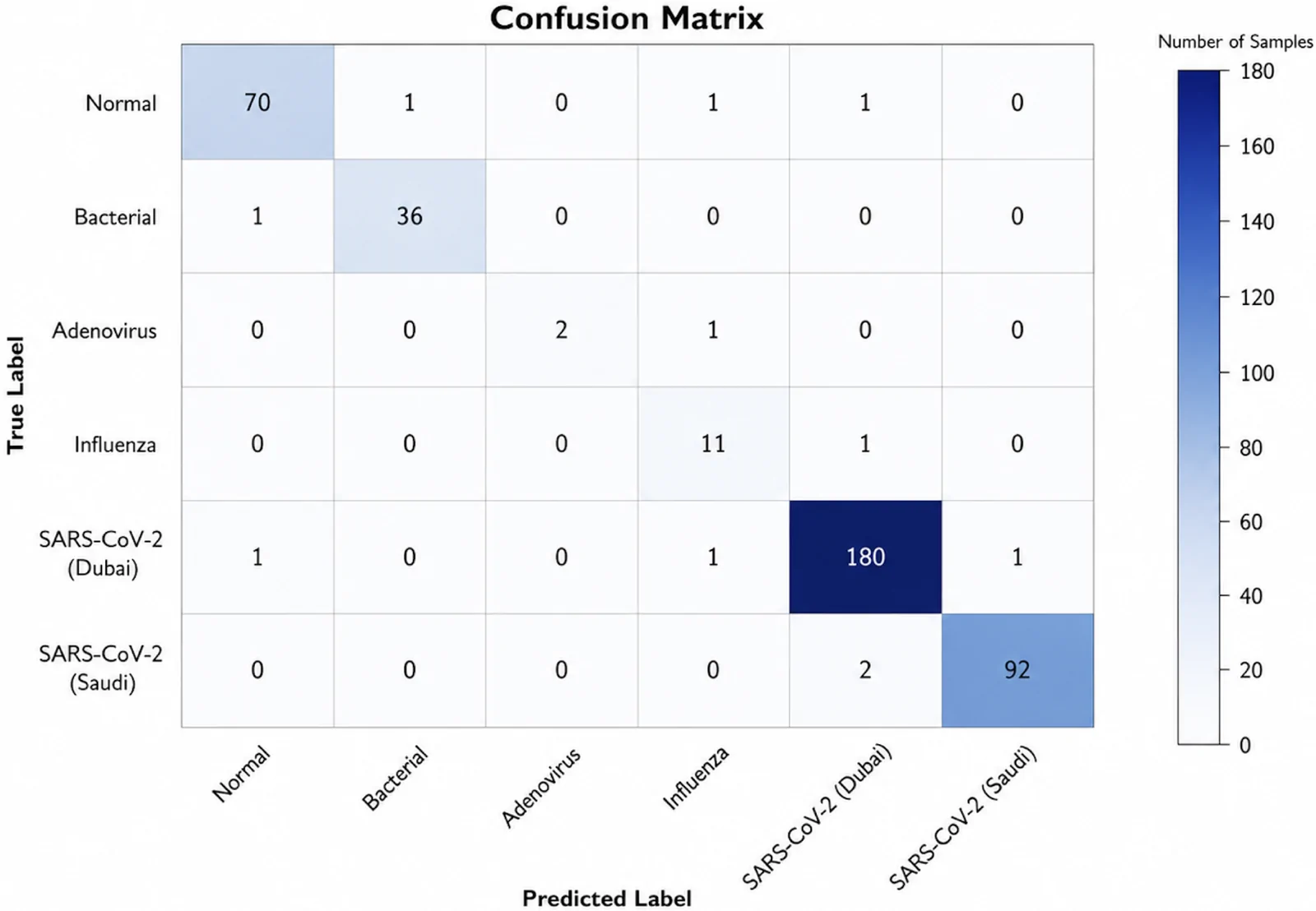
Confusion matrix of the proposed multimodal CNN–MLP fusion framework evaluated on the finalized held-out test set containing 402 samples across six diagnostic categories.

To evaluate classification behavior across disease categories, a confusion matrix was computed using the finalized held-out test set, as shown in Figure 8. The finalized test set contains 402 samples across six diagnostic categories, matching the test partition reported in Table 5. The results demonstrate strong classification performance across both majority and minority categories, with most predictions concentrated along the diagonal, indicating stable multimodal discrimination across heterogeneous respiratory disease classes.

Although limited misclassification was observed between several clinically overlapping categories, the overall confusion matrix behavior suggests balanced multimodal learning performance without substantial bias toward dominant classes.

### 5.3 Class-wise performance analysis

The class-wise performance metrics and confusion matrix were updated to ensure full consistency with the finalized held-out test partition summarized in Table 5. Because the MERS-CoV category contains only a single original image and is represented exclusively within the training subset, MERS-CoV is not included in the validation or test-set evaluation metrics.

Table 6 summarizes the class-wise precision, recall, F1-score, and support values corresponding to the finalized held-out test set containing 402 samples.

**Table 6:** Class-wise precision, recall, F1-score, and support values for diagnostic categories represented in the finalized held-out test set.

Class	Precision	Recall	F1-Score	Support
Normal	0.97	0.96	0.96	73
Bacterial	0.97	0.97	0.97	37
Adenovirus	1.00	0.67	0.80	3
Influenza	0.79	0.92	0.85	12
SARS-CoV-2 (Dubai)	0.98	0.98	0.98	183
SARS-CoV-2 (Saudi)	0.99	0.98	0.98	94
Overall Accuracy				**0.97**

MERS-CoV is excluded because the dataset contains only one original image, which is represented exclusively within the training subset.

Bold values indicate the total number of samples across all diagnostic categories.

### 5.4 Ablation and modality contribution analysis

To further investigate the contribution of individual framework components, additional comparative analysis was conducted across different modality and architectural configurations. Specifically, the proposed multimodal CNN–MLP fusion framework was evaluated relative to image-only CNN models, clinical-data-only MLP models, and alternative multimodal learning baselines discussed throughout the manuscript.

The image-only CNN branch demonstrated strong radiographic feature extraction capability consistent with prior CXR-based diagnostic frameworks. However, its performance remained more sensitive to overlapping radiographic patterns and minority-class imbalance. Similarly, the clinical-data-only MLP branch captured meaningful patient-specific contextual information, including symptoms, laboratory findings, and demographic variables, but lacked direct radiographic representation necessary for robust pneumonia discrimination across heterogeneous disease categories.

In contrast, the proposed multimodal fusion framework consistently demonstrated stronger overall diagnostic stability and more balanced performance across complementary evaluation metrics. Integrating radiographic image representations with structured clinical embeddings enabled the framework to jointly leverage modality-specific strengths while mitigating limitations associated with isolated unimodal learning.

Furthermore, the imbalance-aware optimization strategy, including class weighting, augmentation, SMOTE, dropout regularization, and early stopping, contributed to improved convergence stability and enhanced minority-class robustness under highly skewed disease distributions.

Although exhaustive module-level ablation experiments were not fully performed for every architectural component because of computational and dataset limitations associated with multimodal deep learning training, the comparative unimodal and multimodal analyses collectively support the contribution of multimodal fusion and imbalance-aware optimization toward the observed performance improvements.

## 6 Discussion

The proposed CNN–MLP multimodal framework was evaluated using a dataset of 2,907 samples comprising both CXR images and structured clinical data. All competing approaches were evaluated under identical experimental conditions using the same 85%/15% train–test split to ensure fair and controlled comparison.

Although the proposed framework achieved a 1.3 percentage point improvement in overall accuracy compared to the multimodal baseline reported by Althenayan et al. [14], the observed improvement should be interpreted within the broader context of multimodal medical classification under severe dataset imbalance and heterogeneous clinical conditions. Importantly, the performance gains were not limited to overall accuracy alone, but were also reflected across sensitivity, F1-score, confusion matrix behavior, validation stability, and minority-class robustness. Furthermore, all models were evaluated using identical preprocessing procedures and dataset partitions to support fair comparative assessment.

While repeated *k*-fold cross-validation and formal statistical hypothesis testing would provide additional statistical rigor, such analyses remain computationally challenging for large multimodal deep learning pipelines involving heterogeneous imaging and clinical modalities. Future work will therefore extend the evaluation using repeated validation protocols and multicenter datasets to further assess statistical robustness and generalizability.

The contribution of the proposed framework should not be interpreted as the introduction of a fundamentally new deep learning paradigm. Rather, the primary contribution lies in the optimization and stabilization of multimodal learning under highly imbalanced and heterogeneous healthcare conditions while maintaining computational efficiency and practical deployability within virtual hospital environments. Although broader comparison against fully reproducible multimodal state-of-the-art frameworks remains difficult because of differences in datasets, imaging modalities, preprocessing pipelines, and evaluation protocols, the proposed framework is now more explicitly contextualized relative to established image-based architectures such as CheXNet and COVID-Net within the broader medical AI literature.

### 6.1 Comparative performance analysis

The study first evaluated traditional machine learning classifiers operating on multimodal feature representations. In this setting, deep radiographic features were extracted from CXRs using a CNN backbone and concatenated with 632 preprocessed clinical variables before being provided to classical classifiers. Logistic Regression achieved 89% accuracy with 81% sensitivity, while Random Forest achieved 83% accuracy and 74% sensitivity. XGBoost and Gradient Boosting demonstrated comparatively stronger performance, achieving 90% and 92% accuracy with sensitivities of 86% and 88%, respectively. Support Vector Machines achieved 85% accuracy with 83% sensitivity. Although Gradient Boosting yielded the strongest performance among traditional machine learning approaches, these methods remained limited by their reliance on fixed feature representations and reduced capacity to model complex nonlinear interactions between radiographic patterns and heterogeneous clinical variables. The performance comparison is summarized in Table 7.

**Table 7:** Performance comparison of classical machine learning models with multimodal feature fusion.

Model	Accuracy (%)	Sensitivity (%)
Logistic regression	89	81
Random forest	83	74
XGBoost	90	86
Gradient boosting	92	88
Support Vector Machine (SVM)	85	83

Beyond classical machine learning approaches, more advanced multimodal deep learning architectures were evaluated to benchmark against state-of-the-art fusion strategies. An attention-based multimodal CNN achieved 88% accuracy and 85% sensitivity, while a multimodal transformer employing cross-attention mechanisms achieved 89% accuracy with the same sensitivity level. Although these architectures improved representational learning compared to simpler machine learning approaches, they did not outperform the strongest ensemble-based classical methods and introduced substantially higher computational complexity without proportional diagnostic gains.

In contrast, the proposed CNN–MLP fusion architecture demonstrated superior performance across all evaluated metrics. The framework achieved 97% accuracy and 97% sensitivity, corresponding to an approximate 5% improvement over the strongest traditional machine learning baseline and an 8%–9% improvement over the evaluated attention-based and transformer-based multimodal models. The architecture combines hierarchical CNN-based radiographic feature extraction with structured clinical encoding through an MLP, followed by late multimodal fusion and dropout regularization. This design enables effective multimodal representation learning while maintaining architectural simplicity, computational stability, and practical deployability. The comparative results are summarized in Table 8.

**Table 8:** Performance comparison of deep learning multimodal approaches.

Model	Accuracy (%)	Sensitivity (%)
Attention-based multimodal CNN	88	85
Multimodal transformer	89	85
Proposed CNN–MLP fusion model	**97**	**97**

Bold values indicate the results achieved by the proposed VHealth-MFusion framework.

### 6.2 Architectural design considerations

Selection of the three-layer MLP architecture was guided by empirical architectural exploration and practical training considerations. Shallower architectures were less effective in modeling nonlinear interactions among heterogeneous clinical variables, whereas deeper MLP configurations increased model complexity and training instability without providing consistent improvement in validation performance. The adopted three-layer architecture therefore represented a balanced compromise between representational capacity, regularization, and computational efficiency for multimodal clinical fusion.

The use of late multimodal fusion was particularly important because CXR images and structured clinical variables differ substantially in dimensionality, representation characteristics, and noise distribution. Independent modality-specific encoding prior to fusion enabled more specialized feature learning while reducing instability associated with premature cross-modal interaction in highly heterogeneous medical data.

Similarly, the selection of a lightweight custom CNN architecture reflected a practical balance between feature extraction capability, computational efficiency, multimodal training stability, and dataset scale. Although larger pretrained architectures may provide stronger generalized radiographic representations, they also introduce substantially greater parameter complexity and increased overfitting risk in relatively limited multimodal medical datasets.

The proposed framework also demonstrated balanced performance across both dominant and minority disease categories under highly imbalanced dataset conditions, suggesting stable multimodal learning behavior and improved robustness beyond the dominant classes.

### 6.3 Multimodal learning and clinical integration

A key advantage of the MLP-based clinical branch is its ability to model nonlinear dependencies among heterogeneous clinical variables within a unified latent representation space. Rather than relying on isolated feature analysis, the framework jointly learns interactions among symptoms, laboratory findings, demographic characteristics, and physiological measurements that collectively contribute to respiratory disease classification.

Although the proposed late-fusion framework jointly learns from imaging and structured clinical information, the current architecture does not explicitly expose how individual clinical variables are weighted relative to radiographic findings during prediction. Instead, multimodal interactions are learned implicitly within the fused latent representation space. Incorporating attention-based fusion and explainability-oriented mechanisms in future work may therefore provide more interpretable insight into modality contribution and feature importance during clinical decision making.

From an ablation perspective, the observed multimodal performance gains appear to arise from the complementary interaction between radiographic image representations and structured clinical feature embeddings rather than from any single isolated architectural component alone. The CNN branch contributes hierarchical radiographic feature extraction, while the MLP branch provides clinically contextualized latent representations derived from symptoms, laboratory findings, and demographic variables. The late-fusion strategy then enables joint multimodal reasoning across heterogeneous healthcare modalities. In addition, imbalance-aware optimization strategies contributed to convergence stability and improved minority-class robustness under highly skewed dataset conditions.

From a broader methodological perspective, the proposed framework extends beyond traditional image-only diagnostic architectures such as CheXNet and COVID-Net by integrating structured clinical variables alongside radiographic imaging within a unified multimodal learning pipeline. While CheXNet and COVID-Net demonstrated strong performance for pneumonia and COVID-19 image classification using CXR imaging alone, they were not designed to jointly model heterogeneous patient-specific clinical information. In contrast, *VHealth-MFusion* was specifically developed to support multimodal diagnostic reasoning within virtual hospital environments where imaging findings are interpreted together with demographic variables, laboratory measurements, vital signs, and clinical observations.

### 6.4 Training stability and overfitting considerations

Because the proposed multimodal framework integrates high-dimensional clinical variables with radiographic image features, controlling overfitting represented an important design consideration. The combined use of dropout regularization, early stopping, augmentation, and class-weighted optimization contributed to stable convergence behavior and reduced the likelihood that the framework merely memorized patient-specific patterns or dataset noise.

The high dimensionality of the tabular branch also introduced potential redundancy among clinical variables. Incorporating preprocessing steps to remove highly correlated and low-information features improved computational efficiency and helped stabilize multimodal training by reducing unnecessary feature overlap within the MLP encoder.

Handling incomplete clinical records represented another important preprocessing consideration because real world healthcare datasets frequently contain sparse or partially missing patient information. Applying consistent statistical imputation and feature-reduction strategies improved data usability while supporting stable multimodal learning under heterogeneous clinical conditions.

A potential concern in highly imbalanced medical datasets is that oversampling techniques such as SMOTE may cause models to overfit minority-class noise, particularly when rare disease categories contain limited original samples. Several observations suggest that substantial overfitting was mitigated in the present study. First, the training and validation curves exhibited relatively stable convergence behavior without severe divergence. Second, minority-class results did not exhibit unrealistic perfect classification behavior; for example, the Adenovirus and MERS-CoV classes retained non-perfect precision and recall values despite imbalance-aware optimization. Third, the proposed framework combined SMOTE with complementary regularization strategies, including dropout, augmentation, early stopping, and class weighting, rather than relying exclusively on synthetic oversampling. Nevertheless, because certain disease categories remain extremely underrepresented, the findings for rare classes should still be interpreted cautiously and validated further using larger multicenter datasets.

### 6.5 Clinical relevance and virtual hospital deployment

By integrating radiographic imaging with structured clinical information, the proposed framework has the potential to support clinicians in rapidly diagnosing pneumonia, particularly within underserved or remote healthcare environments where access to specialists may be limited. Such capabilities may contribute to faster triage and treatment decision making within virtual hospital ecosystems.

The framework demonstrates particular promise for deployment within virtual hospital settings that require rapid remote diagnostic support under resource-constrained conditions. However, additional investigation through large-scale multicenter clinical evaluation remains necessary before real world deployment readiness can be fully established.

Reliable confidence estimation and predictive calibration are especially important in virtual hospital environments because clinical decision-support systems must communicate not only predictions, but also the associated uncertainty of those predictions. While the current framework demonstrated strong multimodal classification performance, formal probability calibration analysis was not performed in the present study. Nevertheless, the softmax output layer provides probabilistic predictions that can support future calibration evaluation. Future work will therefore investigate uncertainty quantification and calibration assessment techniques, including reliability diagrams, Expected Calibration Error (ECE), and temperature scaling, to further improve deployment reliability and clinical interpretability.

Generalization across healthcare institutions also remains an important challenge for multimodal medical AI systems because imaging quality, clinical workflows, patient populations, and structured data distributions may vary substantially across centers. Although the proposed framework demonstrated stable performance under the evaluated experimental setting, the current study did not include formal external multicenter validation or leave-one-institution-out evaluation protocols. Therefore, the present findings should primarily be interpreted as evidence of controlled multimodal framework feasibility rather than conclusive proof of universal cross-institution deployment readiness.

### 6.6 Interpretability, fairness, and methodological considerations

Interpretability remains a critical requirement for trustworthy deployment of multimodal AI systems within clinical and virtual hospital environments. The primary focus of the current study was placed on evaluating multimodal fusion performance, robustness under class imbalance, and stable multimodal learning under heterogeneous healthcare conditions. Consequently, formal interpretability validation experiments, such as Grad-CAM visualization for radiographic attention analysis and SHAP/LIME-based attribution analysis for structured clinical variables, were not included within the present experimental scope.

Although the current framework produces probabilistic predictions through multimodal feature integration, the absence of explicit interpretability analysis limits the ability to directly verify alignment between learned model behavior and established clinical reasoning. Accordingly, the findings should currently be interpreted primarily as evidence of multimodal diagnostic feasibility rather than fully interpretable clinical decision-support readiness.

Fairness and demographic generalizability also remain important considerations for multimodal healthcare AI systems. Although detailed demographic breakdown statistics cannot be publicly released because of institutional and privacy restrictions associated with the dataset, the inclusion of demographic variables within the multimodal framework supports broader contextualized clinical learning across heterogeneous patient characteristics. Future work will further investigate subgroup-specific performance analysis and fairness-aware evaluation across demographic populations using larger multicenter datasets.

The proposed framework additionally relies on diagnostic labels inherited from the original multimodal dataset curation process. Although detailed annotation workflow metadata cannot be publicly disclosed because of institutional restrictions, maintaining consistent label definitions across both the baseline and proposed models supports fair comparative evaluation under identical dataset conditions.

From a broader methodological perspective, the novelty of the proposed framework lies primarily in the practical integration, optimization, and stabilization of multimodal learning under highly constrained virtual hospital conditions. Specifically, the framework addresses several operational challenges simultaneously, including severe class imbalance, incomplete structured clinical data, multimodal heterogeneity, computational efficiency, and multimodal convergence stability within remote healthcare diagnostic workflows.

Finally, a major strength of the present study is that comparative evaluation was performed under dataset-consistent conditions using the same multimodal dataset and equivalent partitioning strategy as the baseline work of Althenayan et al. [14]. This reduces potential evaluation bias and supports a more reliable assessment of the methodological contribution introduced by the proposed CNN–MLP multimodal fusion framework.

## 7 Limitations

Despite the strong multimodal classification performance achieved by the proposed *VHealth-MFusion* framework, several limitations remain.

One important limitation is the extreme underrepresentation of certain rare disease categories within the dataset, particularly MERS-CoV, which contains only a single original image sample. Because this class is absent from the validation and test subsets, reliable class-specific evaluation for MERS-CoV cannot be established. Accordingly, MERS-CoV was retained only to preserve the original dataset taxonomy and clinical heterogeneity, while all class-specific performance claims related to MERS-CoV were removed from the revised results.

Another limitation is that the study relied on a single curated multimodal dataset without external multicenter validation or leave-one-institution-out evaluation. Consequently, the broader generalizability of the proposed framework across heterogeneous healthcare systems, imaging acquisition settings, and patient populations remains an important area for future investigation.

Although all comparative models were evaluated under identical experimental settings and demonstrated consistent multimodal performance trends across multiple evaluation metrics, repeated cross-validation, formal statistical significance testing, confidence interval estimation, and bootstrapping analyses were not performed due to the computational complexity associated with multimodal deep learning training. Future studies should incorporate repeated validation protocols and larger multicenter datasets to provide stronger statistical characterization of model robustness, uncertainty, and reproducibility.

Another limitation is that detailed subgroup fairness analysis across demographic populations was not performed. Future research should incorporate fairness-oriented validation protocols and demographic-specific evaluation to better assess robustness across diverse patient populations and healthcare environments.

The current study also did not include formal interpretability validation experiments, such as Grad-CAM visualization or SHAP/LIME-based feature attribution analysis. While the proposed framework demonstrated strong predictive performance, additional explainability analysis will be important to further evaluate alignment between model decision behavior and clinical reasoning.

In addition, exhaustive module-level ablation analysis was not performed for every architectural component due to computational constraints associated with multimodal deep learning training. Future work should further investigate the isolated contributions of individual fusion, regularization, and imbalance-handling mechanisms.

Another limitation is that direct comparison with external multimodal state-of-the-art frameworks remains challenging because many published studies rely on different datasets, imaging modalities, preprocessing strategies, class definitions, and evaluation protocols. Therefore, cross-study numerical comparisons should be interpreted cautiously.

Finally, detailed information regarding the dataset annotation workflow could not be publicly disclosed due to NDA and institutional privacy restrictions associated with the underlying clinical dataset.

## 8 Conclusion and future work

This study presented *VHealth-MFusion*, a hierarchical multimodal deep learning framework that integrates chest X-ray imaging with structured clinical data for pneumonia diagnosis. The proposed pipeline combines CNN-based radiographic feature extraction with clinical tabular encoders within a unified multimodal learning architecture trained using imbalance-aware strategies. Under the evaluated experimental setting, the proposed framework achieved an accuracy of 97.2%, outperforming the hierarchical multimodal CNN baseline reported by Althenayan et al. [14], which achieved 95.9% accuracy on the same dataset.

The findings demonstrate that multimodal fusion improves diagnostic robustness compared to image-only and earlier multimodal approaches. In particular, integrating structured clinical information alongside radiographic features improves the framework’s ability to capture complementary diagnostic patterns within clinically imbalanced scenarios. These results further highlight the importance of combining imaging and patient-related information to better reflect real world clinical diagnostic workflows.

Beyond predictive performance, this work contributes a modular multimodal learning framework designed to support reproducibility and facilitate adaptation to other clinical contexts. The proposed architecture also demonstrates how AI-driven multimodal diagnostic systems can support the broader vision of virtual hospital ecosystems, where remote triage and diagnosis increasingly depend on the integration of heterogeneous clinical information across distributed healthcare environments.

Future work will focus on evaluating pretrained medical imaging backbones, including ResNet and EfficientNet architectures, to investigate whether richer pretrained radiographic representations can further improve multimodal generalization and robustness. Additional research will explore transformer-based multimodal architectures to support more advanced end-to-end joint learning across imaging and clinical modalities.

Future studies will also investigate probabilistic calibration and uncertainty-aware multimodal learning approaches to improve confidence estimation and support safer deployment in clinical decision-support environments. Expanding the framework through larger multicenter datasets, additional imaging sources, and more comprehensive patient information will also be important for improving robustness and external generalizability.

Finally, future work will incorporate explainability mechanisms, including saliency-based visualization and SHAP-based feature attribution analysis, to enhance clinical transparency, interpretability, and trustworthiness in real world virtual healthcare deployment settings.

## Data Availability

The data analyzed in this study is subject to the following licenses/restrictions: The dataset used in this study is proprietary and cannot be made publicly available due to restrictions imposed by a Non-Disclosure Agreement (NDA) signed by the authors. Access to the dataset may be considered upon reasonable request and subject to the terms of the NDA, institutional approvals, and applicable ethical regulations. Researchers interested in accessing the dataset for research purposes may contact the corresponding author at Salsalamah@vt.edu to discuss potential access conditions. All requests will be evaluated in accordance with the restrictions outlined in the NDA and relevant institutional policies. Requests to access these datasets should be directed to Salsalamah@vt.edu.
